# Energy Expenditure during Sexual Activity in Young Healthy Couples

**DOI:** 10.1371/journal.pone.0079342

**Published:** 2013-10-24

**Authors:** Julie Frappier, Isabelle Toupin, Joseph J. Levy, Mylene Aubertin-Leheudre, Antony D. Karelis

**Affiliations:** 1 Department of Kinanthropology, Université du Québec à Montréal, Montreal, Quebec, Canada; 2 Department of Sexology, Université du Québec à Montréal, Montreal, Quebec, Canada; 3 School of Public Health, Université de Montréal, Montreal, Quebec, Canada; University of Bath, United Kingdom

## Abstract

**Objective:**

To determine energy expenditure in kilocalories (kcal) during sexual activity in young healthy couples in their natural environment and compare it to a session of endurance exercise.

**Methods:**

The study population consisted of twenty one heterosexual couples (age: 22.6 ± 2.8 years old) from the Montreal region. Free living energy expenditure during sexual activity and the endurance exercise was measured using the portable mini SenseWear armband. Perceived energy expenditure, perception of effort, fatigue and pleasure were also assessed after sexual activity. All participants completed a 30 min endurance exercise session on a treadmill at a moderate intensity.

**Results:**

Mean energy expenditure during sexual activity was 101 kCal or 4.2 kCal/min in men and 69.1 kCal or 3.1 kCal/min in women. In addition, mean intensity was 6.0 METS in men and 5.6 METS in women, which represents a moderate intensity. Moreover, the energy expenditure and intensity during the 30 min exercise session in men was 276 kCal or 9.2 kCal/min and 8.5 METS, respectively and in women 213 kCal or 7.1 kCal/min and 8.4 METS, respectively. Interestingly, the highest range value achieved by men for absolute energy expenditure can potentially be higher than that of the mean energy expenditure of the 30 min exercise session (i.e. 306.1 vs. 276 kCal, respectively) whereas this was not observed in women. Finally, perceived energy expenditure during sexual activity was similar in men (100 kCal) and in women (76.2 kCal) when compared to measured energy expenditure.

**Conclusion:**

The present study indicates that energy expenditure during sexual activity appears to be approximately 85 kCal or 3.6 kCal/min and seems to be performed at a moderate intensity (5.8 METS) in young healthy men and women. These results suggest that sexual activity may potentially be considered, at times, as a significant exercise.

## Introduction

Health professionals are starting to recognize that sexual activity in humans could be an important aspect on their overall health and quality of life since this activity is practiced regularly by most individuals throughout their lifetime [1-6]. However, due to the intimate and sensitive nature of sexuality, few studies have investigated if sexual activity could be considered as an exercise which involves a significant amount of energy expenditure [7-14]. For example, in 1966, Masters and Johnson [12] were one of the first authors to examine the physiological responses of sexual activity albeit in a laboratory setting. The authors reported 11 years of observational studies that involved 382 female volunteers, 18 to 78 years of age, and 312 male volunteers, 21 to 89 years of age. The authors observed a progressive increase in respiratory rates as high as 40 respirations per minute, an increase in heart rate as high as 110 to 180 beats/min and an increase in systolic blood pressure from 30 to 80 mm Hg during sexual activity. In 1970, Hellerstein and Friedman [9] investigated sexual activity in middle-aged men (mean age 47.5 years) with their wives using 24-hour ambulatory electrocardiogram (ECG) monitors. The mean heart rate at the time of orgasm was 117.4 beats per minute with a range of 90 to 144 beats per minute. Of particular interest was their finding on peak coital heart rate, which was usually lower than the heart rates achieved with normal daily activities (mean of 120.1 beats per minute). In 1984, Bohlen et al. [8] studied 10 married couples in a laboratory setting using ECG, oxygen consumption (measured using a fast responding polarographic O_2_ gas analyzer), heart rate and blood pressure monitoring before and during 4 types of sexual activity: self-stimulation, partner stimulation, man-on-top and woman-on-top coitus. Results from men aged 25 to 43 years old showed that sexual activity with their partners had minimal effect on heart rate, oxygen consumption and blood pressure during foreplay and stimulation (sexual activity before the onset of orgasm). That is, self-stimulation increased heart rate by 37 % from baseline to orgasm compared with a 51 % increase with man-on-top coitus. Not surprisingly, orgasm was associated with maximal increases in all three of these parameters. The highest intensity was associated with coitus, especially the man-on-top position, where 3 to 4 METS were exerted. In 2007, Palmeri et al. [14] reported that in 19 men and 13 women aged 40-75 years old, the intensity of sexual activity was comparable to stage II of the standard multistage Bruce protocol (moderate intensity) on a treadmill for men and stage I (low intensity) for women. In addition, maximal heart rate and blood pressure during sexual activity was approximately 75 % of that attained during maximum treadmill stress testing of the Bruce protocol. Collectively, based on these above studies, the physiological responses of sexual activity seem to be at a moderate intensity. 

It should be noted that all of the above studies that were conducted were limited to simple techniques such as heart rate and blood pressure for the determination of intensity during sexual activity. It is also important to indicate that sexual activity is a non-steady-state activity. Thus, the heart rate-blood pressure relationship may not remain linear during sexual activity, suggesting that these physiological parameters might not be primary indicators for the measurement of energy expenditure and/or intensity for this type of activity. In addition, these studies had important environmental and methodological limitations. That is, the experiments, in general, were performed in the laboratory rather than in the couple’s usual and natural environment (i.e. home). Moreover, the equipment that was used to measure exertion in these studies could have affected the ability to perform a sexual activity (i.e. mask placed on the mouth for the measurement of oxygen consumption as well as electrodes, cuffs and cables placed on the body during sexual intercourse). Taken together, performing these studies in the laboratory setting and the potential interferences due to the equipment used, minimize the chances to re-enact the normal intimacy observed in real-life conditions. Furthermore, most of the framework of knowledge of the physiological responses during sexual activity was gathered in studies that were conducted more than a quarter of a century ago. Thus, the conclusions that were made in these older studies may not be as representative in our modern times due to the wider acceptance of sexuality in today’s society. Also, the previous studies used couples that had a wide range of age and did not report the differences between ages from both individuals in the couple, which could be a confounding factor (younger vs. older individuals). Finally, no study, to our knowledge, has investigated the amount of energy expenditure in kilocalories (kcal) during sexual activity and has compared this energy expenditure, using the same subjects, to a regular exercise which could provide valuable clinical information to health professionals. There is even a myth which suggests that energy expenditure during sexual activity is between 100 to 300 kcal per session for each individual involved [15]. However, no scientific data has documented this claim. Collectively, new timely studies on energy expenditure and sexual activity in a natural environment without any methodological interference are needed in order have more conclusive findings. Therefore, the purpose of the present study was to determine energy expenditure in kilocalories as a primary outcome and intensity (METS) as a secondary outcome using a simple, non-obstructive and accurate method for the measurement of energy expenditure (SenseWear armband) during sexual activity in young healthy couples and compare it to a session of endurance exercise. This objective will provide us with new insights on the potential role of sexual activity as a physical exercise. In addition, as a tertiary outcome, we investigated the perception of energy expenditure, fatigue, effort, pleasure and appreciation of the couples after sexual activity using a questionnaire. Our primary goal was not to examine the physiological responses (i.e. heart rate and blood pressure) of sexual activity *per se* but to get a better understanding of the energy expenditure in kilocalories, which is a unit that is more commonly used today by health professionals and the general public for health purposes. 

## Methods

### Participants

Twenty one heterosexual couples from the Montreal region were recruited between September 2012 to April 2013 for this study. Volunteers were recruited from the Université du Québec à Montreal and from the Montreal population. To be included in the study, participants had to meet the following criteria: 1) aged between 18-35 years old, 2) born in the province of Quebec and francophone, 3) Caucasian, 4) non-sedentary (>2 hours a week of structured exercise), 5) no sexual dysfunctions, 6) be sexually active (at least one sexual activity per week), 7) in a loving, monogamous and stable relationship with their partner for a duration between 6 and 24 months, and 8) the use of oral contraception for women. In addition, all participants reported no cardiovascular diseases, diabetes or any orthopaedic limitations. Body weight was measured using an electronic scale (Balance Industrielles, Montreal, Canada) and standing height was measured using a wall stadiometer (Perspective Enterprises, Michigan, USA). Body mass index [BMI = body weight/Height (m^2^)] was calculated. All procedures were approved by the Ethics Committee of the Université du Québec à Montréal. Participants were fully informed about the nature, goal, procedures and risks of the study, and gave their informed consent in writing. 

### Endurance Exercise Session

All participants completed one endurance exercise session at the start of the study which consisted of a 5 min warm-up (walking) followed by 30 minutes of exercise on a treadmill (0 % grade) at ~65 % of maximal heart rate, which represents a moderate intensity and ended with a 5 min cool down. The modalities of the endurance exercise session were based on the recommendations of the American College of Sports Medicine and the American Heart Association which recommends 30 min of exercise at a moderate intensity 5 times a week to the general population [16]. Our goal was to choose a form of exercise that could be regularly practised by the general population and to be used as a control.

### Sexual Activity

In the present study, sexual activity was defined as the onset of foreplay, intercourse and at least one orgasm by either the man or woman and ended at the couple’s discretion. During a one month period, couples were instructed to perform one sexual activity per week in their homes. Thus, all couples had performed a total of 4 sexual activities. The couples were instructed to perform their usual sexual activities and not to use any drugs, alcohol or medication for erectile dysfunction (i.e. Viagra) before the sexual activity as well as not to perform any paraphilic sexual activities. 

### Questionnaire

All participants completed a questionnaire after each sexual activity. The following seven questions were asked: 1) How would you compare your effort between sexual activity and that of the exercise performed on the treadmill? 2) What was your perception of fatigue after sexual activity? 3) What was your perception of energy expended after sexual activity? 4) What was your personal perception of pleasure after sexual activity? 5) What was your partner’s perception of pleasure after sexual activity? 6) What was your appreciation of the sexual activity in comparison to that of the exercise performed on the treadmill? and 7) How many Calories did you think you burned during sexual activity? The participants had a choice of three categories (i.e. low, medium or high) to choose from for the first six questions. As for the seventh question, participants were asked to write down a number. The couples were instructed not to share or discuss their information with each other. In the present study, we presume that the perception of sexual activity is unique for each participant. Thus, the aim of these questions was to explore in detail how participants are making sense of their personal experiences. 

### Measure of Energy Expenditure

Free living energy expenditure during sexual activity and the endurance exercise session was measured using the portable mini SenseWear armband (Bodymedia, Pittsburgh, PA). The portable armband uses a 3-axis accelerometer, a heat flux sensor, a galvanic skin response sensor, a skin temperature sensor, and a near-body ambient temperature sensor to capture data. These data as well as body weight, height, handedness and smoking status (smoker or non-smoker) are used to calculate energy expenditure. The armband was placed on the upper left arm (on the triceps at the mid-humerus point) of each volunteer. The net output is a measure of energy expenditure (kcal) and intensity (METS) utilized by the participant across time. Data analysis for energy expenditure and intensity were available minute by minute (SenseWear professional software 7.0). This method of energy expenditure measurement has been validated by several studies and is known to be 92 % accurate compared to the gold standard method of doubly labeled water [17-24]. In addition, a test–retest reliability trial (n = 34) for energy expenditure performed in our laboratory showed an intra-class correlation coefficient of 0.97 [25]. It should be noted that mean energy expenditure and intensity were measured during the four different sessions of sexual activity. Couples were instructed to wear the armbands right before the start of the sexual activity and take it off at the end of their sexual encounter.

### Statistical Methods

Results are presented as means ± SD. Normality was verified using the Kurtosis-test. To address our primary and secondary outcomes, paired t-tests were used to identify differences in energy expenditure and intensity between sexual activity and the 30 minute exercise session in both men and women. Moreover, a repeated measures ANOVA using the post-hoc assessment of LSD was used to detect differences in energy expenditure between all four sessions of sexual activity in both men and women. Furthermore, unpaired t-tests were used to detect differences in age, body mass index, energy expenditure and intensity between men and women. To address our tertiary outcome, 1) paired t-tests were used to identify differences in measured energy expenditure and perceived energy expenditure in both men and women and 2) a Chi-square test was performed to analyze differences in perception of effort, fatigue, appreciation and pleasure in men and women. Statistical analysis was performed using SPSS 20 for Windows (Chicago, IL). Significance was accepted at *p* < 0.05.

## Results

Mean duration of the relationship of the couples was 13.4 ± 5.5 months (range: 6-24 months) and the mean duration of the sexual activity was 24.7 ± 12.2 min (range: 10-57 min). BMI, absolute and relative energy expenditure during sexual activity, as well as absolute and relative energy expenditure during treadmill exercise were significantly higher in men compared to women (Table 1). When statistically controlling for BMI, significant differences in absolute and relative energy expenditure during sexual activity between men and women persisted. No differences in age, perceived energy expenditure, intensity during sexual activity and intensity during the treadmill exercise were observed between men and women (Table 1). In addition, no differences in percentage (absolute and relative) of the energy expenditure and intensity during sexual activity compared to that of the treadmill exercise were observed between men and women (Table 1). 

**Table 1:** Mean energy expenditure and intensity during sexual activity in men and women.

	**All Participants (n = 42)**	**Men (n = 21)**	**Range**	**Women (n = 21)**	**Range**
Age (yrs)	22.6 ± 2.8	22.7 ± 2.7	19-30	22.4 ± 2.9	19-31
Body mass index (kg/m^2^)	22.9 ± 2.8	24.2 ± 2.7	19.5-31.0	21.6 ± 2.3*	16.9-26.6
Duration of relationship (months)	13.4 ± 5.5				
Duration of SA (min)	24.7 ± 12.2				
AEE of SA (kcal)	84.8 ± 43.5^a^	101 ± 52^a^	13.0-306.1	69.1 ± 25.6*^a^	11.6-164.1
REE of SA (kcal/min)	3.6 ± 1.3^a^	4.2 ± 1.3^a^	1.2-7.9	3.1 ± 1.0*^a^	1.2-8.4
Intensity of SA (METS)	5.8 ± 1.3^a^	6.0 ± 1.3^a^	1.4-9.2	5.6 ± 1.2^a^	1.3-9.6
PEE of SA (kcal)	87.4 ± 54.2	100 ± 63	15.0-300	76.2 ± 43.3	5.0-250
AEE of treadmill (kcal)	245 ± 73	276 ± 68	149-390	213 ± 64.3*	120-381
REE of treadmill (kcal/min)	8.2 ± 2.4	9.2 ± 2.3	5.0-13.0	7.1 ± 2.2*	4.0-12.7
Intensity of treadmill (METS)	8.4 ± 1.6	8.5 ± 1.5	5.7-10.6	8.4 ± 1.8	5.2-10.8
AEE SA vs. AEE of treadmill (%)	37.8 ± 19.9	31.6 ± 11.9	17.2-58.2	44.0 ± 24.2	12.9-98.5
REE SA vs. REE of treadmill (%)	46.7 ± 16.9	47.5 ± 17.5	22.6-87.7	46.0 ± 16.6	16.7-81.9
Intensity SA vs. intensity treadmill (%)	71.0 ± 20.6	73.0 ± 20.1	37.6-114	69.0 ± 21.4	27.4-102

Values are means ± SD. SA: sexual activity; AEE: absolute energy expenditure; REE: relative energy expenditure; PEE: perceived energy expenditure. * Significantly different from men (*P* < 0.05). ^a^ Significantly different from treadmill (*P* < 0.05).

**Figure 1:**
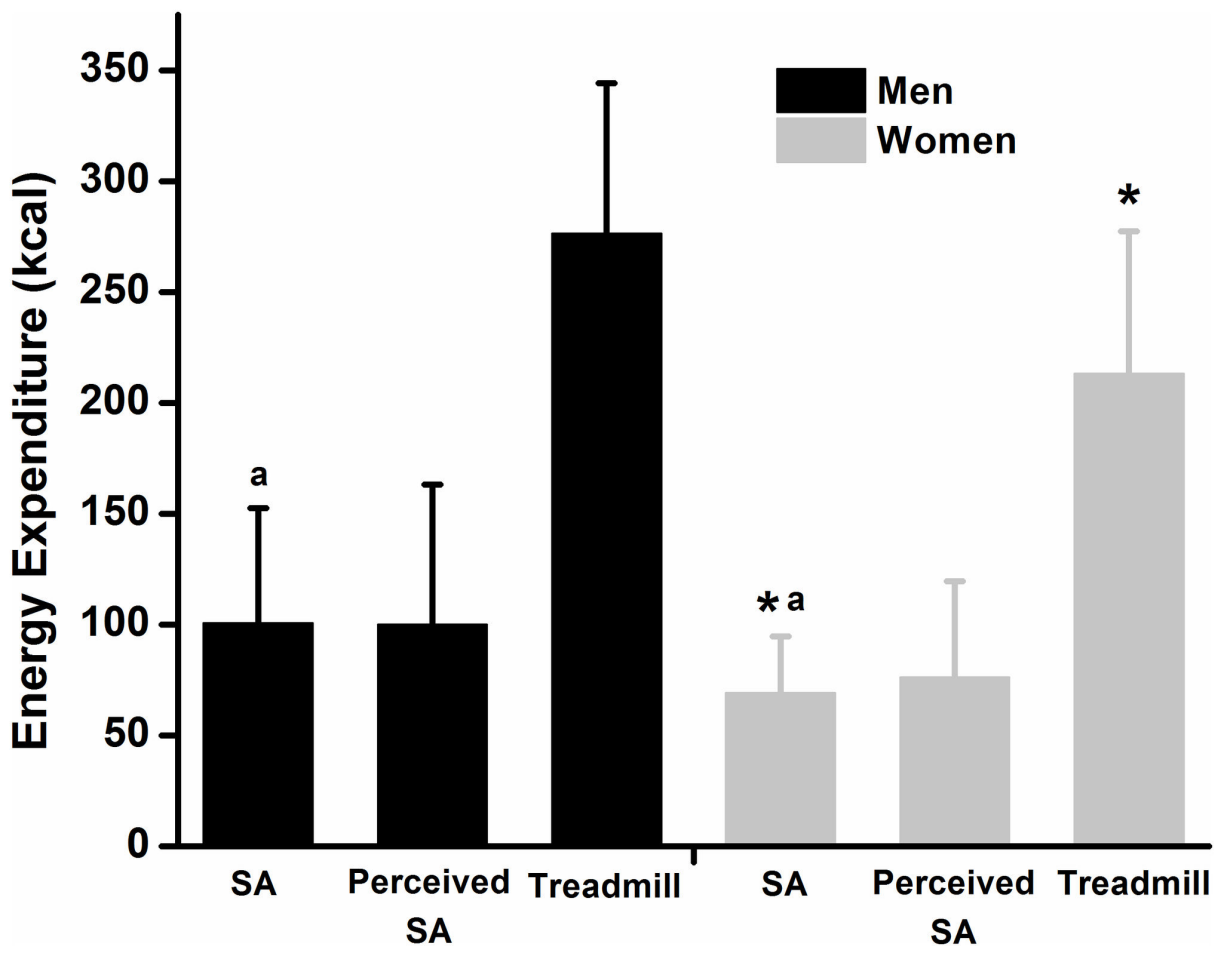
Differences in energy expenditure in all participants as well as in men (black) and women (grey). Values are the mean ± SD. * Significantly different from men (*P* < 0.05). ^a^ Significantly different from treadmill (*P* < 0.05). SA: sexual activity.

**Figure 2:**
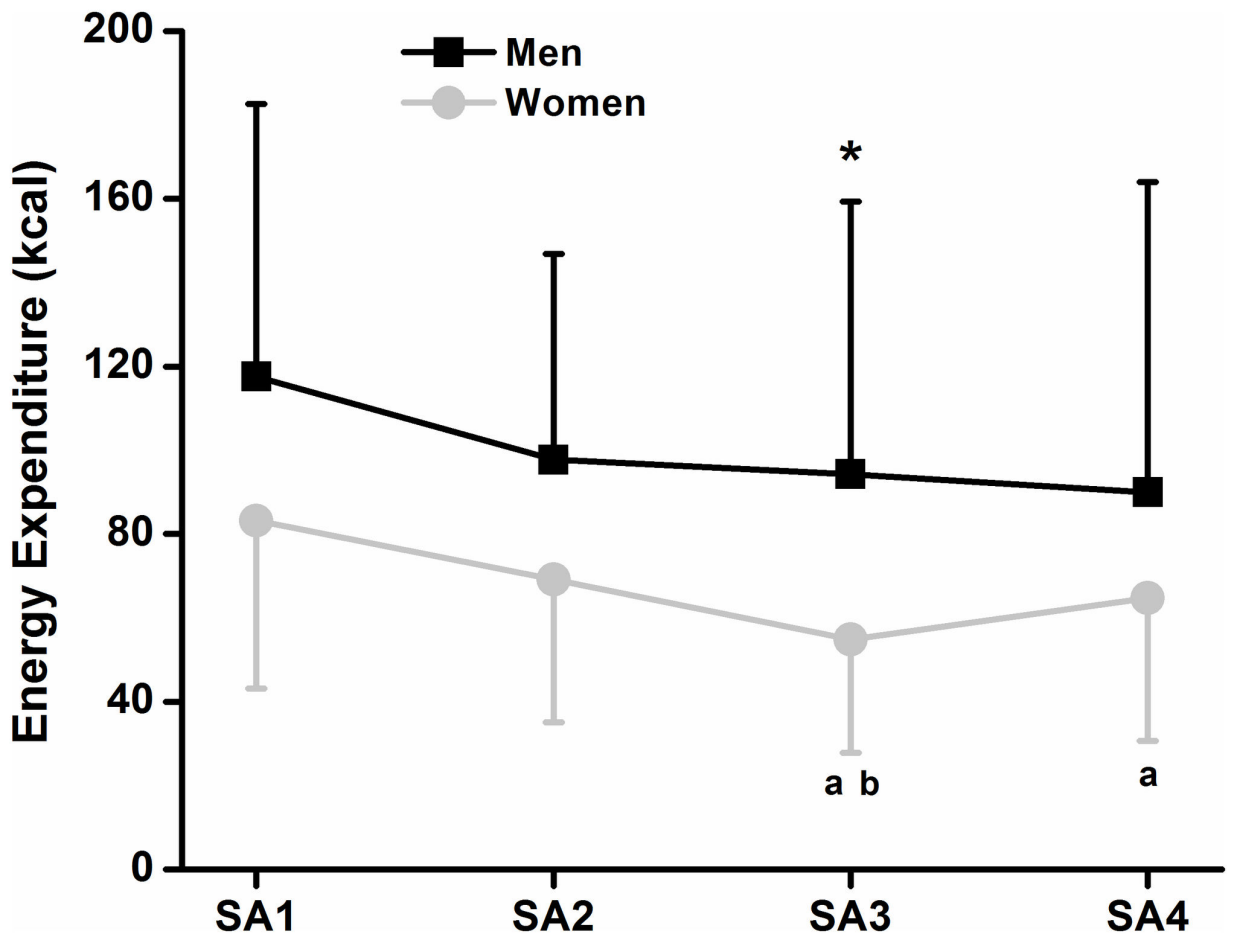
Differences in energy expenditure between all four sessions of sexual activity in men (black) and women (grey). Values are the mean ± SD. * Significantly different from the first session in men (*P* < 0.05). ^a^ Significantly different from the first session in women (*P* < 0.05). ^b^ Significantly different from the second session in women (*P* < 0.05). SA: sexual activity.

Figure 1 and Table 1 show mean energy expenditure, intensity and perceived energy expenditure during sexual activity as well as mean energy expenditure and intensity during the 30 min treadmill exercise for all of the participants. We also investigated absolute energy expenditure during sexual activity across all four time points in men (118 ± 65 vs. 98 ± 49 vs. 94 ± 65 vs. 90 ± 74 kcal) and women (83 ± 40 vs. 69 ± 34 vs. 55 ± 27 vs. 65 ± 34 kcal). We observed that energy expenditure in session three was significantly lower than session one in men and women. Also in women, energy expenditure was significantly lower in session four when compared to session one and in session three when compared to session two (Figure 2). These results could suggest that men and women present different patterns of energy expenditure during sexual activity across time. We also calculated the equivalence in percentage of the energy expenditure and intensity during sexual activity with that of the energy expenditure of the treadmill exercise. Results show that absolute and relative energy expenditure as well as intensity during sexual activity represented ~38 %, 47 % and 71 % of the energy expenditure (absolute and relative) and intensity of the treadmill exercise, respectively. Ranges for energy expenditure, intensity and perceived energy expenditure for the sexual activity as well as the treadmill exercise are also presented in table 1. 

Table 2 shows the perception of effort, fatigue, appreciation, energy expended as well as personal and the partner’s pleasure after sexual activity in all participants as well as men and women. Briefly, only 5 % of all participants reported that sexual activity was more strenuous when compared to the 30 min treadmill exercise. Nineteen percent of all participants reported that they were highly fatigued and 7 % reported a high energy expended after sexual activity. Approximately 98 % of all participants reported that sexual activity was more pleasant than the 30 min treadmill exercise. Finally, 81 % reported a high level of personal pleasure and ~79 % reported a high level of their partner’s pleasure after sexual activity. No differences between men and women in all of the above parameters were noted. 

**Table 2:** Perception of effort, fatigue, appreciation and pleasure after the sexual activity in men and women.

	**All Participants (n = 42)**	**Men (n = 21)**	**Women (n = 21)**
**Comparison of Effort Between Sexual Activity and Treadmill**			
Less strenuous (%)	57.5	55.0	60.0
Comparable (%)	37.5	35.0	40.0
More strenuous (%)	5.0	10.0	0.0
**Perception of Fatigue**			
Slightly Fatigue (%)	42.9	38.1	47.6
Moderately Fatigue (%)	38.1	38.1	38.1
Highly Fatigue (%)	19.0	23.8	14.3
**Perception of Energy Expended**			
Low (%)	28.6	19.0	38.1
Medium (%)	64.3	66.7	61.9
High (%)	7.1	14.3	0.0
**Perception of Appreciation of Sexual Activity Compared to Treadmill**			
Less pleasant (%)	0.0	0.0	0.0
As pleasant (%)	2.5	0.0	5.0
More pleasant (%)	97.5	100.0	95.0
**Perception of Personal Pleasure**			
Low (%)	0.0	0.0	0.0
Medium (%)	19.0	19.0	19.0
High (%)	81.0	81.0	81.0
**Perception of Partner’s Pleasure**			
Low (%)	0.0	0.0	0.0
Medium (%)	21.4	28.6	14.3
High (%)	78.6	71.4	85.7

## Discussion

Sexual activity is an important and relevant activity to human life and appears to impact on the mental, physical and social health as well as on the quality of life of the individual [1,4-6]. In the next decade, we will continue to see advances in the field of human sexuality and its relationship with overall health. Considering that sexual activity may be one of the most regularly practiced activities throughout an individual’s life time, it seems important to conduct research on this topic. Therefore, the the purpose of the present study was to examine the energy expenditure during sexual activity in young healthy individuals in their natural environment and compare it to a session of endurance exercise. 

Results of the present study show that mean energy expenditure during sexual activity was 101 kcal or 4.2 kcal/min in men and 69 kcal or 3.1 kcal/min in women. Energy expenditure from sexual activity was significantly higher in men compared to women. In addition, mean intensity was 6.0 METS in men and 5.6 METS in women, which represents a moderate intensity according the American College of Sports Medicine (ASCM) (moderate: 3-6 METS) [26]. Furthermore, by comparison, the level of intensity that is exerted from sexual activity could be higher to that of walking at 4.8 km/h but lower to that of jogging at 8 km/h [27]. The level of intensity of sexual activity in the present study may give health professionals a better understanding on the potential risk for myocardial infarction in cardiac patients since this topic appears to be a preoccupation in the field of medicine [28]. Moreover, the energy expenditure and intensity during the 30 min exercise session in men was 276 kcal or 9.2 kcal/min and 8.5 METS, respectively and in women 213 kcal or 7.1 kcal/min and 8.4 METS, respectively. Both energy expenditure and intensity were significantly higher during the 30 min exercise session than the sexual activity. Interestingly, the highest range value achieved by men for absolute energy expenditure during sexual activity can potentially be higher than that of the mean absolute energy expenditure of the 30 min exercise session (i.e. 306 vs. 276 kcal, respectively), whereas this was not observed in women. Also, we noted that the highest range value of intensity in both men and women could be higher when compared to the mean intensity of the 30 min exercise session (9.2 vs. 8.5 METS; 9.6 vs. 8.4 METS, respectively). Finally, it should be noted that the absolute and relative energy expenditure of sexual activity in the present study represented more than one third of the absolute and relative energy expenditure of the 30 min treadmill exercise session, respectively. However, the intensity level during sexual activity represented more than two thirds of the intensity of the treadmill exercise. Taken together, these results suggest that sexual activity may potentially be considered, at times, as a significant exercise. 

Results of the present study are slightly different from two other studies in which only the level of intensity can be compared [8,14]. That is, in the study of Bohlen et al. [8] the level of intensity during sexual activity (especially the man-on-top position) was between 3 to 4 METS in young (average: 33 years) Caucasian men, whereas in the present study intensity was 6.0 METS in men. In the study of Palmeri et al. [14], the intensity of sexual activity was comparable to stage II of the standard multistage Bruce protocol on a treadmill for men (average: 55 years), which represents a moderate intensity and is similar to the results in the present study for men. However, for women (average: 51 years) sexual activity was comparable to stage I, which represents a low intensity and differs with the results of the present study for women which showed a moderate intensity. 

Perception of energy expenditure, effort, fatigue, appreciation and pleasure of the participants were also measured. Interestingly, perceived energy expenditure was similar in men (100 kcal) and in women (76 kcal) when compared to measured energy expenditure. This suggests that the perception of energy expenditure at the group level appears to be accurate during sexual activity. Moreover, a very small minority of participants reported that sexual activity was more strenuous when compared to the treadmill exercise and almost a fifth reported to be highly fatigued after sexual activity. Furthermore, almost all of the participants reported that sexual activity was more pleasant than the treadmill exercise and that most men and women reported a high level of personal pleasure and of the partner’s pleasure during sexual activity. Thus, health professionals may want to consider sexual activity as part of their planning of intervention programs for a healthy lifestyle. It should be noted that no differences between men and women were observed for perception of energy expenditure, effort, fatigue, appreciation and pleasure.

There are several limitations in the present study. First, since our cohort is composed of sexually active young healthy men and women, thus, our findings are limited to this population. Further research should be performed in other populations and age groups. Second, the characteristics of the cohort reflect strict inclusion and exclusion criteria. However, our results are strengthened by using a simple, non-obstructive and accurate method for the measurement of free living energy expenditure in the couple’s natural environment making this study more representative.

## Conclusions

The present study indicates that energy expenditure during sexual activity appears to be approximately 85 kcal or 3.6 kcal/min and seems to be performed at a moderate intensity in young healthy men and women. These results suggest that sexual activity may potentially be considered, at times, as a significant exercise. Moreover, both men and women reported that sexual activity was a highly enjoyable and more appreciated than the 30 min exercise session on the treadmill. Therefore, this study could have implications for the planning of intervention programs as part of a healthy lifestyle by health care professionals. Future studies may want to examine the relationship between psychosocial/qualitative factors with sexual activity and energy expenditure which could explain how these variables could affect overall health and quality of life.
